# Epitope mapping of PfCP-2.9, an asexual blood-stage vaccine candidate of *Plasmodium falciparum*

**DOI:** 10.1186/1475-2875-9-94

**Published:** 2010-04-12

**Authors:** Changling Li, Rui Wang, Yuan Wu, Dongmei Zhang, Zhicheng He, Weiqing Pan

**Affiliations:** 1 Department of Pathogenic Biology, Second Military Medical University, 800 Xiang Yin Road, Shanghai 200433, China

## Abstract

**Background:**

Apical membrane antigen 1 (AMA-1) and merozoite surface protein 1 (MSP1) of *Plasmodium falciparum *are two leading blood-stage malaria vaccine candidates. A *P. falciparum *chimeric protein 2.9 (PfCP-2.9) has been constructed as a vaccine candidate, by fusing AMA-1 domain III (AMA-1 (III)) with a C-terminal 19 kDa fragment of MSP1 (MSP1-19) via a 28-mer peptide hinge. PfCP-2.9 was highly immunogenic in animal studies, and antibodies elicited by the PfCP-2.9 highly inhibited parasite growth *in vitro*. This study focused on locating the distribution of epitopes on PfCP-2.9.

**Methods:**

A panel of anti-PfCP-2.9 monoclonal antibodies (mAbs) were produced and their properties were examined by Western blot as well as *in vitro *growth inhibition assay (GIA). In addition, a series of PfCP-2.9 mutants containing single amino acid substitution were produced in *Pichia pastoris*. Interaction of the mAbs with the PfCP-2.9 mutants was measured by both Western blot and enzyme-linked immunosorbent assay (ELISA).

**Results:**

Twelve mAbs recognizing PfCP-2.9 chimeric protein were produced. Of them, eight mAbs recognized conformational epitopes and six mAbs showed various levels of inhibitory activities on parasite growth *in vitro*. In addition, seventeen PfCP-2.9 mutants with single amino acid substitution were produced in *Pichia pastoris *for interaction with mAbs. Reduced binding of an inhibitory mAb (mAb7G), was observed in three mutants including M62 (Phe^491^→Ala), M82 (Glu^511^→Gln) and M84 (Arg^513^→Lys), suggesting that these amino acid substitutions are critical to the epitope corresponding to mAb7G. The binding of two non-inhibitory mAbs (mAbG11.12 and mAbW9.10) was also reduced in the mutants of either M62 or M82. The substitution of Leu^31 ^to Arg resulted in completely abolishing the binding of mAb1E1 (a blocking antibody) to M176 mutant, suggesting that the Leu residue at this position plays a crucial role in the formation of the epitope. In addition, the Asn^15 ^residue may also play an important role in the global folding of PfCP-2.9, as its substitution by Arg lead to reduced binding of most mAbs and abolishing the binding of mAb6G and mAbP5-W12.

**Conclusions:**

This study provided valuable information on epitopes of PfCP-2.9 vaccine candidate through generation of a panel of mAbs and a series of PfCP-2.9 mutants. The information may prove to be useful for designing more effective malaria vaccines against blood-stage parasites.

## Background

Malaria remains a severe tropical disease. It causes about 247 million cases in the world and nearly a million deaths in 2006, most of which were in children under five years of age in sub-Saharan Africa [1]. The *Plasmodium *parasite of malaria has a complex life cycle, and the blood stage is believed to be responsible for the disease and directly associated with clinical manifestations and therefore considered as a primary target for developing malaria vaccines.

Apical membrane antigen 1 (AMA-1) and merozoite surface protein 1 (MSP1) of *Plasmodium falciparum *play essential roles in the blood stage of malarial life cycle and are two promising vaccine candidates [2,3]. After a similar proteolytic processing step by *P. falciparum *subtilisin-like protease 2 (PfSUB2) [4,5], their C-terminal regions remain anchored within the parasite membrane, and then are carried into the newly invaded red blood cells [6,7]. *Plasmodium falciparum *chimeric protein 2.9 (PfCP-2.9) constructed by fusing PfAMA-1 (III) with PfMSP1-19 in previous studies [8,9], was shown to enhance the product yield, immunogenicity, and antibody-mediated *in vitro *growth inhibition assays (GIA). When formulated with Montanide ISA720, the vaccine was also confirmed to be safe, tolerable and immunogenic in two Phase I clinical trials [10,11]. Although higher levels of antibody titers are usually associated with better protection, some researchers argued that fine specificity of antibodies was closely associated with biological functions [12-14]. As described by Uthaipibull *et al *[13], antibodies to PfMSP1-19 could be classified as inhibitory (inhibiting secondary processing and/or erythrocyte invasion), blocking (blocking the function of inhibitory antibodies) and neutral (neither inhibitory nor blocking). It was proposed that there may also exist malaria specific non-AMA-1 blocking antibodies, because it was found that a fraction of malaria specific immunoglobulins (IgGs) from Malians that did not bind to AMA-1 substantially reduced the growth-inhibitory activity of both total IgGs and anti-AMA-1 antibodies from the sera of U.S. vaccinees [15]. Therefore, fine epitope mapping of the PfCP-2.9 protein is needed for further development of the vaccine candidate.

Some epitopes of PfMSP1-19 have been described previously, especially since the discovery of the three-dimensional structure of the molecule [16-19]. PfMSP1-19 consists of two epidermal-growth-factor (EGF)-like domains, forming a disc-like shaped molecule. Inhibitory monoclonal antibodies (mAbs), including 12.8 and 12.10, recognize conformational epitopes on one side of the molecule, while blocking or neutral mAbs seem to recognize epitopes localized on the other side. The difference in distribution of the inhibitory and neutral epitopes is consistent with the formation of a ring structure between PfMSP1-19 and both 2F10 (neutral) and 12.10 (inhibitory) antibodies [12]. The structure of PfAMA-1 (III) has also been resolved, although its functional epitopes remain unknown [20].

It was found in a previous study [9] that PfCP-2.9 maintained the important conformational epitopes on PfMSP1-19 through interaction with a panel of conformational mAbs and displayed elevated biological activity compared with the two components. However, functional epitopes on PfCP-2.9 remain unclear. In this study, a panel of anti-PfCP-2.9 mAbs and a series of mutated proteins with single amino acid substitution on PfCP-2.9 were prepared to clarify the distribution of epitopes on PfCP-2.9.

## Methods

### Generation of hybridomas and production of mAbs to PfCP-2.9

A panel of mAbs to the PfCP-2.9 were produced in this study by the hybridoma technology as previously described [21]. Briefly, BALB/c mice were immunized by administration of 50 μg PfCP-2.9 in the presence of FCA (Freund's complete adjuvant)/FIA (Freund's incomplete adjuvant), followed by three booster injections of the same dose at 15-day intervals. Spleen cells were separated from mice 3 days after the last immunization and fused with SP2/0 mouse myeloma cells. The hybrids were then selected in hypoxanthine-aminopterin-thymidine (HAT) medium and cloned by limiting dilution. For large-scale production, hybridoma cell lines were abdominally injected into mice to generate mAbs in ascites. The mAbs were further purified by protein G affinity chromatography, dialyzed against RPMI 1640 media, aliquoted and stored at -80°C.

### Parasite cultures and GIA

*Plasmodium falciparum *FCC1/HN strain was maintained and used for the assay [9]. Synchronized parasites were cultured until the mature trophozoite stage of development. 100 μl culture containing 2% RBC with a 0.5% ± 0.1% initial parasitemia and 25 μl of test mAbs was added to 96-well flat-bottomed plates (Greiner Bio-one, Germany). The initial concentrations of the test mAbs were adjusted to 2 mg/ml before the assay. IgG of rabbit anti-AMA1-C1 (2 mg/ml) was used as an inhibitory control (gift of NIH) and mAb5.2 was used as a non-inhibitory control. All the mAbs were sterilized by filtration through a 0.22 μm Millex-GV filter (Millipore, USA). After 42-h incubation with the purified antibody, 50 μl well-mixed parasites from each assay well were dispensed into 0.25 ml cold phosphate buffered saline (PBS, pH 7.4) in a fresh 96-well U-bottomed ELISA microplate (Greiner Bio-one, Germany). The plates were loaded into a Centrifuge 5810R (Eppendorf, Germany) and centrifuged at 1,300 × *g *for 10 min at 4°C. A portion (0.24 ml) of the supernatant was removed from each well, and the plate was frozen at -80°C for 30 min to lyse the pelleted cells. After the plate was brought back to room temperature (RT), 0.1 ml complete pLDH substrate (100 mM Tris-HCl pH 7.5, 50 mM sodium L-lactate, and 0.25% Triton X-100 containing 0.2 mg nitroblue tetrazolium ml^-1^, 0.05 mg 3-acetylpyridine adenine dinucleotide ml^-1^, and 1 U Diaphorase ml^-1^) was added to each well. The plate was kept in the dark and read at 15 and 30 min. The absorbance of wells in each plate was measured at 650 nm on a Microplate reader (ELx800 ELISA reader; Bio-Tek Instruments, Winooski, VT), and the percentage of inhibition was calculated as 100 - (A_650 _of the test sample - A_650 _of the RBC only)/(A_650 _of mAb5.2 - A_650 _of RBC only) ×100.

### Mutagenesis

The *pfcp-2.9 *gene [9] was mutated by site-directed mutagenesis using *Pichia *optimized codons by overlap extension polymerase chain reaction (PCR) [22], based on the rule of "safe substitution" [23], which would minimally affect the global folding of the target protein. The constructed plasmid vector of *pfcp-2.9*/pBluescriptKS was amplified by KOD-plus DNA polymerase (Toyobo, Osaka, Japan) with the mutant primers listed in Table 1. Then the mutated genes were transformed into competent *Escherichia coli *DH5α cells (Life technologies). After screening by analysis of restriction enzyme digests, the sequence of selected mutant clones was confirmed using an Applied Biosystems ABI 377 DNA sequencer according to the manufacturer's instructions.

**Table 1:** Primers for preparation of mutant genes by site-directed mutagenesis

Number	Designation	Directions	Primer sequences (5' to 3')
01	M17		
		Forward	CCATGTTCATTATTTAAAGATG
		Reverse	CCTTCATAATTTCATCTTTAAATAATGAAC
02	M18		
		Forward	CATGTTCATTATATAGAGATG
		Reverse	CATCTCTATATAATGAACATG
03	M55		
		Forward	CCGCTGATAATAAAGACTC
		Reverse	CCGGAGTCTTTATTATCAG
04	M56		
		Forward	CCGCTGATGATAGAGACTC
		Reverse	CCGGAGTCTCTATCATCAG
05	M62		
		Forward	CTTTAAAGTGTGCATGTGACCC
		Reverse	GGGTCACATGCACACTTTAAAG
06	M71		
		Forward	GGTTTCACAAACTACATGTCG
		Reverse	CGACATGTAGTTTGTGAAACC
07	M81		
		Forward	CTTTGTTTGTAAATGTATCGAAAGAAG
		Reverse	CTTCTTTCGATACATTTACAAACAAG
08	M82		
		Forward	CGTTTCTTTGTTTGTAAATGTGTCCAAAGAAG
		Reverse	GCCCTTCTTTGGACACATTTACAAACAAAG
09	M84		
		Forward	CGAAAGAAAGGCTGAAG
		Reverse	CTTCAGCCTTTCTTTCG
10	M159		
		Forward	GCAATGTCCAGAAAATTCTG
		Reverse	CCAGAATTTTCTGGACATTG
11	M160		
		Forward	GTCCACAAAGATCTGG
		Reverse	CCAGATCTTTGTGGAC
12	M165		
		Forward	CCGGTTTCAAACATTTAG
		Reverse	CCGCTAAATGTTTGAAAC
13	M172		
		Forward	GATGAAAGAGAATACTGTAAATG
		Reverse	CATTTACAGTATTCTCTTTCATC
14	M174		
		Forward	CCGGAATGTAGATGTTTG
		Reverse	CCGCAAACATCTACATTCTTC
15	M176		
		Forward	GTAAATGTAGGTTGAATTACAAACAAGAAGG
		Reverse	CCTTCTTGTTTGTAATTCAACCTACATTTAC
16	M185		
		Forward	GGTGATAGATGTGTTGAAAATCC
		Reverse	CCGCAACACATCTATCACC
17	M188		
		Forward	GTGTTTTAAATCCACAACC
		Reverse	GGTTGTGGATTTAAAACAC
18	Universal		
		Forward	CCCTCGAGAAAAGAGCTTTGTC
		Reverse	CCGGAATTCCTATTAATGATGATG

### Protein expression and purification

Each mutated 6×His tagged gene was cloned into the expression vector of pPIC9K and linearized via *Sac*I digestion. After transformation into *Pichia pastoris *by electroporation, selection of His^+ ^transformants and G418-resistant clones was performed according to the manufacturer's instructions (Invitrogen, San Diego, CA). The selected clone was grown in minimal glycerol medium at 30°C overnight and then resuspended in buffered methanol-complex medium containing 0.5% methanol to induce expression of the target gene. After 72-h induction, the supernatant was collected by centrifugation at 10,000 × *g *for 10 min, and then dialyzed against lysis buffer (50 mM NaH_2_PO_4_, 300 mM NaCl, pH 8.0) at 4°C overnight. Filtration was performed with a microporous membrane (0.22 μm in diameter) before the protein was purified by Ni-NTA affinity chromotography. Elution was performed with elution buffer (lysis buffer containing 50~250 mM imidazole, pH 8.0) after the column was equilibrated by lysis buffer and washed extensively with wash buffer (lysis buffer containing 10 mM imidazole, pH 8.0).

### Analysis of antibody binding to PfCP-2.9 and mutated proteins by Western blot and ELISA

Purified protein samples were subjected to sodium dodecyl sulfate polyacrylamide gel electrophoresis (SDS-PAGE) after boiling in the sample buffer without a reducing agent. The protein bands were electrophoretically transferred to the nitrocellulose membrane (Huashun, China) by the Trans-blot SD semi-dry transfer cell (Bio-rad, USA). Blots were blocked with Tris-buffered saline (TBS, pH 7.4) containing 3% skim milk (TBS-M) at RT for 1 h; washed with TBS containing 0.05% Tween 20 (TBS-T) for three times and then probed by the primary antibody in TBS-M at RT for 2 h. The blots were washed and incubated with alkaline phosphatase (AP)-conjugated goat anti-mouse IgG (H+L) (Jackson ImmunoResearch, USA) at a 1:1000 dilution for 1 h, followed by three washes in TBS-T and in AP buffer (100 mM NaCl, 5 mM MgCl_2_6H_2_O, 100 mM TrisCl, pH 9.5) for 10 min. Finally, antibody binding was detected by nitrotetrazolium blue chloride (NBT)/5-bromo-4-chloro-3-indolylphosphate (BCIP) (Genview, USA) substrate in AP buffer (66 μl NBT/33 μl BCIP/10 ml AP buffer) and terminated by washing with double distilled water. For qualitative comparison, proteins were analyzed on a SDS-PAGE gel to obtain an equal amount. Antibody binding to PfCP-2.9 and the mutated proteins was compared and scored as follows: "++" indicates approximately the same amount of antibody binding, "+" indicates reduced binding to the mutated protein compared with PfCP-2.9, and "-" indicates no detectable antibody binding to the mutated protein.

ELISA was also performed to examine the difference in binding intensity between PfCP-2.9 and its mutated proteins as described previously [9]. To avoid errors between the plates, PfCP-2.9 and its mutated proteins were coated in the same plate, while comparing with the same mAbs. MAb5.2 was adopted as an internal reference to assure that the proteins were equally coated, as the mapping of mAb5.2 had no overlapping epitope with the substitutions that we synthesized on PfCP-2.9 [12]., The absorbance of PfCP-2.9 for each mAb was used to obtain the percentage of the reduction rate (Rr) for the mutated proteins. The Rr value for the binding of a certain mAb was calculated as 100% - (A_450 _of a mutated protein/A_450 _of PfCP-2.9) ×100%. The recording method was similar to that in Western blot: "++" indicates about the same binding between PfCP-2.9 and mutated proteins with a Rr value within 25%; if the Rr value was between 25% and 75%, a signal of "+" would be marked; finally, "-" was assigned when the Rr value was above 75%, indicating no detectable antibody binding of the mutated protein as compared with PfCP-2.9.

## Results

### Production and characterization of mAbs against PfCP-2.9

To identify epitopes of PfCP-2.9, a total of 12 mAbs recognizing the chimeric protein were prepared and characterized. Determination of binding regions on PfCP-2.9 for each mAb was investigated using recombinant PfAMA-1 protein (NIH) and PfMSP1-19 expressed from *P. pastoris*. The results showed that five mAbs (mAb2G, mAb7G, mAb10G, mAbG11.12 and mAbW9.10) recognized the AMA-1 portion, and four (mAb13, mAb16, mAbW17 and mAbP5-W12) recognized PfMSP1-19 epitopes, while the remaining three (mAb6G, mAb17, mAb18.20) failed to recognize either component of the chimeric protein in Western blot. PfCP-2.9 was further treated to determine whether its recognition by the mAbs was conformation-dependent. The results showed that except for mAb2G, mAb16, mAb17 and mAb18.20, interactions of the rest mAbs were reduction-sensitive. In addition, all the mAbs were detected for their ability to inhibit parasite growth *in vitro *by GIA and the results showed that six mAbs (mAb16, mAb17, mAbW17, mAb7G, mAb10G, and mAb13) had various levels of inhibitory activities from 9~32% The properties of the mAbs are summarized in Table 2.

**Table 2:** Properties of monoclonal antibodies against PfCP-2.9

	Recognition				
Designation	PfCP-2.9	AMA-1	MSP-1_19_	Reduction sensitivity	GIA (%)
mAb2G	+	+	-	-	0
mAb6G	+	-	-	+	0
mAb7G	+	+	-	+	12
mAb10G	+	+	-	+	10
mAbG11.12	+	+	-	+	0
mAb13	+	-	+	+	9
mAb16	+	-	+	-	31
mAb17	+	-	-	-	20
mAb18.20	+	-	-	-	0
mAbW9.10	+	+	-	+	0
mAbW17	+	-	+	+	32
mAbP5-W12	+	-	+	+	0

### Design and production of PfCP-2.9 mutants with single amino acid substitution

**Figure 1:**
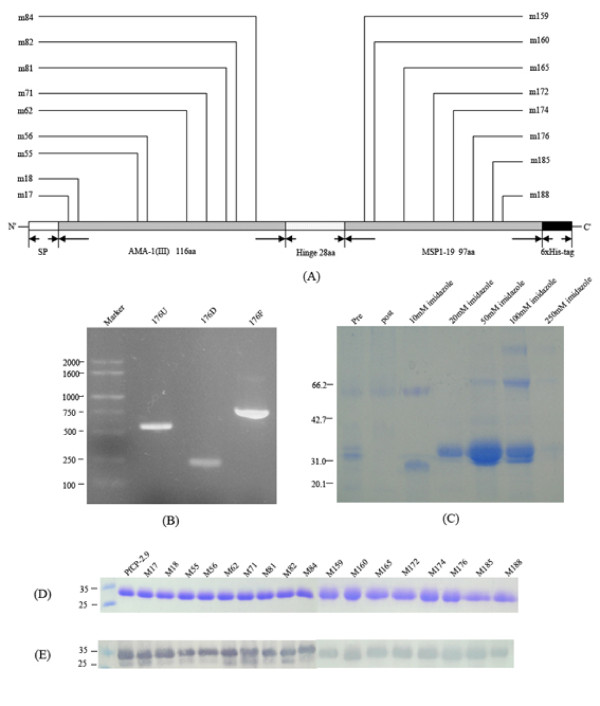
**Production and identification of PfCP-2.9 mutants**. (A) Schematic representation of site-directed mutagenesis of PfCP-2.9. The 17 mutants were designated according to the positions of corresponding mutated amino acid resudues on PfCP-2.9. The N-terminal signal peptide (SP) and C terminal 6×His tag of each construct remained intact, and were involved in subsequent expression and purification, respectively. (B) Assembly of each mutant gene by overlap extension PCR method described in Method section. The 176 U was generated by PCR using the forward universal primer and a reverse mutant primer, while the 176 D fragment generated using the reverse universal primer and a forward mutant primer. The two fragments were combined to generate the entire mutant gene (176F) by PCR using the forward and reverse universal primers. (C) Purification of PfCP-2.9 mutants by Ni-NTA chromotography. The supernatant of culture expressing M176 gene in *P. pastoris *was applied to Ni-NTA agarose columns. Pre- and post-column fraction as well as elution fractions with various concentration of imidazole was analyzed on a 12% SDS-PAGE gel, followed by Coomassie blue staining. (D) SDS-PAGE analysis of purificed PfCP-2.9 mutants. Each lane indicated each purified PfCP-2.9 mutant; (E) Western blot analysis of PfCP-2.9 mutants. Polyclonal rabbit antibodies to the PfCP-2.9 were used as the primary antibody for this detection.

Seventeen mutants of the PfCP-2.9 with single amino acid substitution on either component of AMA-1 (III) or MSP1-19 were produced in *Pichia pastoris*. Figure 1A shows changes in location of the residues. Substitutions on the AMA-1 (III) component were selected as the highly conserved amino acids in different *Plasmodium *species [24] or amino acids within the epitope of mAb F8.12.10, which cross-reacts with several species of the *Plasmodium *[25]. These mutations were designed to discover possible functional epitopes on the AMA-1 (III) component. Some amino acid residues that participated in the formation of definitive blocking epitopes or those surface-exposed on the crystal structure of PfMSP1-19 were also selected to investigate the corresponding epitopes on PfCP-2.9. A general description of the substitutions is shown in Table 3.

**Table 3:** General description of the substitutions made within PfCP-2.9

	Location on PfCP-2.9	Position^b^	Amino acid			
			
Designation^a^			PfCP-2.9	Mutant	Selection	Reference
M17	AMA-1(III)	446	Tyr	Phe	*c*	[24]
M18	AMA-1(III)	447	Lys	Arg	*c*	[24]
M55	AMA-1(III)	484	Asp	Asn	*c*	[24]
M56	AMA-1(III)	485	Lys	Arg	*d*	[25]
M62	AMA-1(III)	491	Phe	Ala	*d*	[25]
M71	AMA-1(III)	500	Ser	Thr	*c*	[24]
M81	AMA-1(III)	510	Val	Ile	*d*	[25]
M82	AMA-1(III)	511	Glu	Gln	*c*	[24]
M84	AMA-1(III)	513	Arg	Lys	*c*	[24]
M159	MSP1-19	14	Gln	Gly	*e*	[19]
M160	MSP1-19	15	Asn	Arg	*f*	[13]
M165	MSP1-19	20	Arg	Glu	*e*	[19]
M172	MSP1-19	27	Glu	Tyr	*f*	[13]
M174	MSP1-19	29	Lys	Arg	*e*	[19]
M176	MSP1-19	31	Leu	Arg	*f*	[13]
M185	MSP1-19	40	Lys	Ile	*e*	[19]
M188	MSP1-19	43	Glu	Leu	*f*	[13]

*a*. Mutated proteins are designated with the corresponding sequence number of the amino acid residues on PfCP-2.9

*b*. Positions for the substitutions numbered from the start of PfAMA-1 (3D7 line) or of PfMSP1-19 (Wellcome/K1 line).

*c*. Highly conserved amino acids across different Plasmodium species.

*d*. Epitopes recognized by mAbF8.12.19.

*e*. Surface-exposed amino acids on the PfMSP1-19 molecule.

*f*. Epitopes recognized by blocking mAbs.

To generate the PfCP-2.9 mutant genes, two overlapping fragments that carried the same point mutation were first produced by PCR amplification using the *pfcp-2.9 *gene as the template and then combined by the second round of PCR. The fragments and the final product of the mutant gene of *m176 *are representatively shown in Figure 1(B). Each PfCP-2.9 mutant was subsequently sequenced before being transformed into *P. pastoris *for expression. The 17 PfCP-2.9 mutants were produced in *Pichia *system in a secreted form at various expression levels. The proteins were then purified by Ni-NTA affinity chromotography. SDS-PAGE and Western blot analyses of the PfCP-2.9 mutant proteins are shown in Figure 1(D) and 1(E), respectively.

### Effects of single amino acid substitution of PfCP-2.9 on mAb binding

**Figure 2:**
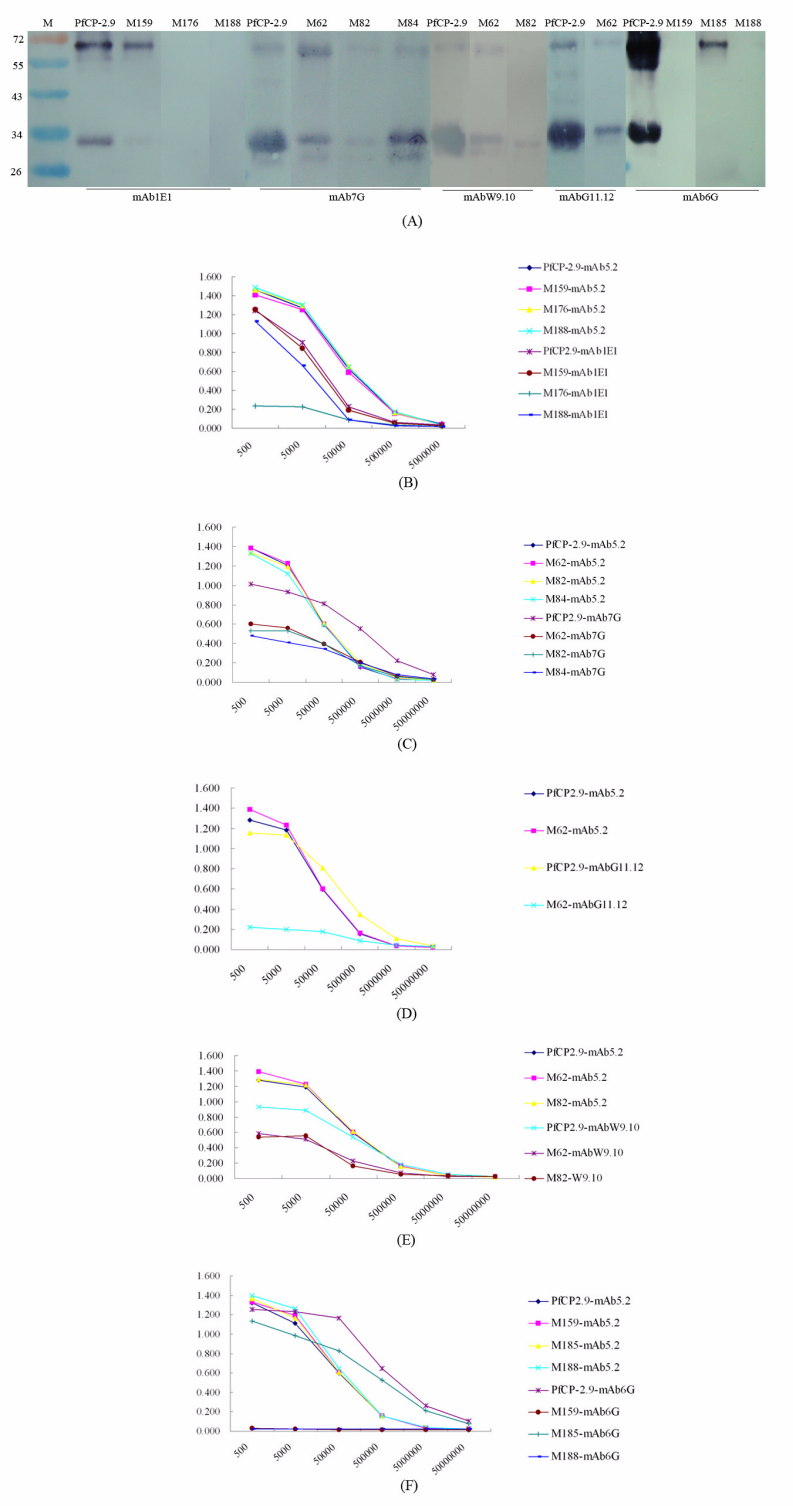
**Western blot and ELISA analysis of interaction of mAbs with the PfCP-2.9 mutants**. (A) Effects of PfCP-2.9 mutant containing a single amino acid substitution on the binding of the mAbs in Western blot. The mAbs indicated were used to investigate the binding differences between PfCP-2.9 and its mutant with a single amino acid substitution. Same amount of each purified protein was subjected to SDS-PAGE and Western blot. As indicated on the gel, reduced binding of the inhibitory mAb7G was observed in the mutants of M62 (Phe^491 ^→ Ala), M82 (Glu^511 ^→ Gln) and M84 (Arg^513 ^→ Lys) compared to the binding of this mAb to the PfCP-2.9. Similarly, the binding of mAbW9.10 was reduced in M62 and M82, seperately. In addition, the binding of mAb1E1 was reduced in M159 (Gln^14 ^→ Gly) and M188 (Glu^43 ^→ Leu), and abolished in M185 (Lys^40 ^→ Ile). The bindings of two non-inhibitory antibodies, mAb6G and mAbP5-W12, were both reduced in M185 (Lys^40 ^→ Ile) and abolished in M159 and M188. (B), (C), (D), (E) and (F): Effects of PfCP-2.9 mutant on the binding of the mAbs in ELISA. Proteins coated were standardized by mAb5.2. The mAbs of 1E1, 7G, G11.12, W9.10 and 6G were serially diluted by PBS-M, followed by a standard ELISA. Their binding differences between PfCP-2.9 and its mutated proteins are shown in (B) to (F). Data shown were repeatable in three separate experiments.

The PfCP-2.9 and its mutants were assayed for mAb binding by both ELISA and Western blot. Epitope mapping of PfCP-2.9 with the mAbs is summarized in Table 4, and the main results of Western blot and ELISA are shown in Figure 2. Mutations in the AMA-1 (III) component of PfCP-2.9 did not change the antibody binding significantly, except that M62 (Phe^491^→Ala) reduced the binding of mAb7G, mAbG11.12 and mAbW9.10; M82 (Glu^511^→Gln) reduced the binding of mAb7G and mAbW9.10, while M84 (Arg^513^→Lys) only reduced the binding of mAb7G. Mutations in the MSP1-19 component of PfCP-2.9 were relatively more informative. Similarly, the binding of mAb6G and mAbP5-W12 was abolished by the replacement of either Gln^14^→Gly (M159) or Glu^43^→Leu (M188) and reduced by the replacement of Lys^40^→Ile (M185). However, they were different in the recognition of PfMSP1-19, i.e. both PfCP-2.9 and PfMSP1-19 were recognized by mAbP5-W12, while only the former was recognized by mAb6G. The substitution of Asn^15^→Arg (M160) reduced the binding of most mAbs and abolished the binding of mAb6G and mAbP5-W12.

**Table 4:** Effects of single amino acid substitutions made within PfCP-2.9 on the binding of monoclonal antibodies

Antigens	Amino acid		Binding of monoclonal antibodies													
	**PfCP-2.9**	**Mutant**	**7G**	**10G**	**13**	**16**	**17**	**W17**	**W9.10**	**G11.12**	**1E1**	**2G**	**6G**	**18.20**	**P5-W12**	5.2

PfCP-2.9			++	++	++	++	++	++	++	++	++	++	++	++	++	++
M17	Tyr	Phe	++	++	++	++	++	++	++	++	++	++	++	++	++	++
M55	Asp	Asn	++	++	++	++	++	++	++	++	++	++	++	++	++	++
M56	Lys	Arg	++	++	++	++	++	++	++	++	++	++	++	++	++	++
M62	Phe	Ala	**+**	++	++	++	++	++	**+**	**+**	++	++	++	++	++	++
M81	Val	Ile	++	++	++	++	++	++	++	++	++	++	++	++	++	++
M82	Glu	Gln	**+**	++	++	++	++	++	**+**	++	++	++	++	++	++	++
M84	Arg	Lys	**+**	++	++	++	++	++	++	++	++	++	++	++	++	++
M159	Gln	Gly	++	++	++	++	++	++	++	++	**+**	++	**-**	++	**-**	++
M160	Asn	Arg	**+**	**+**	**+**	**+**	**+**	**+**	**+**	**+**	++	**+**	**-**	**+**	**-**	++
M165	Arg	Glu	++	++	++	++	++	++	++	++	++	++	++	++	++	++
M172	Glu	Tyr	++	++	++	++	++	++	++	++	++	++	++	++	++	++
M174	Lys	Arg	++	++	++	++	++	++	++	++	++	++	++	++	++	++
M176	Leu	Arg	++	++	++	++	++	++	++	++	**-**	++	++	++	++	++
M185	Lys	Ile	++	++	++	++	++	++	++	++	++	++	**+**	++	**+**	++
M188	Glu	Leu	++	++	++	++	++	++	++	++	**+**	++	**-**	++	**-**	++

"++": strong binding; "**+**": reduced binding; "**-**": no binding

The bindings of the mAbs to PfCP-2.9 and its mutants in ELISA were largely in agreement with the results of the Western blot, with only one exception that the binding of mAb1E1 was abolished by M176 and reduced by M188 in ELISA; however, it was abolished by M176 or M188 and reduced by M159 in Western blot. Mutations that abolished binding of the mAbs in immunoblot had an Rr value over 75%; those that reduced binding in the immunoblot had an Rr value between 25% and 75%, and those that had no effect on the binding in the immunoblot had an Rr value below 25%.

## Discussion

PfCP-2.9 is a promising vaccine candidate among many worthy vaccines under development for prevention of malaria. It is made by fusing two blood-stage antigens (PfAMA-1 (III) and PfMSP1-19) through a hinge. Although animal experiments have demonstrated that this vaccine is much more immunogenic than either component or the mixture of the two components, its functional epitopes remain unclear.

To identify epitopes of PfCP-2.9, a panel of 12 mAbs were prepared in the present study, of which six showed various levels of inhibitory activity as identified by GIA. The Western blot analysis showed that most of the inhibitory mAbs were sensitive to reduction, indicating that the disulfide-bond stabilized conformation is still critical for the chimeric protein, which is consistent with the previous work [9]. Interestingly, three of the mAbs only recognized PfCP-2.9, but not either component. Possibility of novel epitopes created by interaction of the two components was excluded through analysis of the nuclear magnetic resonance (NMR) solution structure of PfCP-2.9 [26]. The specificity of the mAbs needs to be further investigated.

**Figure 3:**
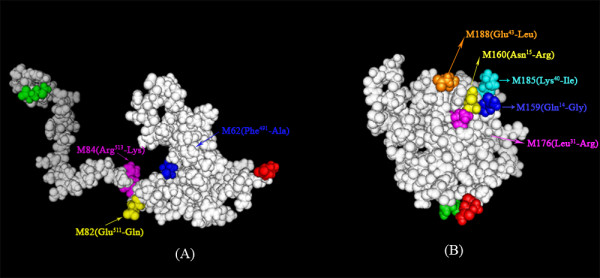
**Localization of functional epitopes of PfCP-2.9 within the three-dimensional structure of AMA-1 (III) and MSP1-19**. The epitopes of PfCP-2.9 were plotted onto the published solution structure of AMA-1 (III) [PDB: 1HN6] [20] and PfMSP1-19 [PDB: 1cej] [18], separately. The N- and C-terminal ends are colored in red and green, respectively. (A) The substitutions affecting the binding of an inhibitory mAb, mAb7G, lie within the C-terminal unstructured region of AMA-1 (III), in close proximity to each other in conformation. (B) The substitutions affecting the binding of non-inhibitory mAb6G and mAbP5-W12 cluster on one of the two sides of the disc-like-shape molecule of PfMSP1-19; while the amino acids involved in the epitope of blocking mAb1E1 are on the same side of the molecule, as reported by Uthaipibull *et al*. [13]. Substitution of Asn^15 ^→ Arg affected the global folding of PfCP-2.9 and is colored in yellow. The figure was created using the PyMOL software [27].

Epitope mapping was carried out by comparing mAb binding to PfCP-2.9 and its mutant proteins through Western blot and ELISA. Apparent binding changes are shown in Figure 2 and the epitopes associated with the changes were plotted onto the published three-dimensional structure of PfAMA-1(III) [PDB: 1HN6] [20] and PfMSP1-19 [PDB: 1cej] [18] by the PyMOL software (DeLano Scientific, San Carlos, CA, USA) [27] as shown in Figure 3. The published structures of the individual components were used for the plotting analysis based on the fact that NMR solution structures of the individual components of PfCP-2.9 were not changed.

These findings may have the following implications:

(1) Of the six mAbs whose binding was reduced or abolished by the mutations synthesized, only mAb7G exibited a mild inhibitory effect. This inhibitory epitope of mAb7G was found on the AMA-1 (III) component. The binding of mAb7G was reduced by any one of the three substitutions (M62: Phe^491^→Ala, M82: Glu^511^→Gln andM84: Arg^513^→Lys). These three amino acids were all surface-exposed and clustered in the C-terminal unstructured region of AMA-1 (III), in close proximity to each other in conformation. The amino acid of Phe^491 ^was involved in an epitope recognized by mAbF8.12.19, which was not inhibitory but involved in interspecies cross-reactivity [25]. Besides the direct steric blockage of AMA-1 function such as displayed by mAb4G2 [28], cross-linking and the consequent inhibition of AMA-1 dispersion across the parasite surface have also been linked to invasion inhibition [28,29]. The mutation of M62 also disrupted the formation of a six-residue ring structure formed by Cys^490^-Cys^507^, Cys^492^-Cys^509^, Phe^491 ^and Arg^508^, which may be of functional importance on AMA-1 (III) [20]. These findings suggest that the amino acid of Phe^491 ^is tightly associated with the formation of the inhibitory epitope of mAb7G. Glu^511 ^and Arg^513 ^are both highly conserved amino acids across different *Plasmodium *species [24], and may also contribute to the formation of the epitope. The binding of mAbG11.12 was reduced by M62 or M82, while the binding of mAbW9.10 was only reduced by M62. Therefore, there is possibility that mAbG11.12 and mAbW9.10 could block the inhibitory effect of mAb7G by competing at the position of both Phe^491 ^and Glu^511 ^and at the position of Phe^491^, respectively.

(2) Some information about blocking epitopes was obtained. It was found that four definitive amino acids (Asn^15^, Glu^27^, Leu^31 ^and Glu^43^) were involved in the formation of blocking epitopes on MSP1-19. It was reported [13] that mutation of these amino acids abolished the binding of blocking mAbs of mAb7.5, mAb2.2, mAb1E1 and mAb111.4, respectively. ELISA was performed to detect mAb1E1 binding to PfCP-2.9 mutant, and the results showed that its binding was abolished by mutant M176 (Leu^31^→Arg) and reduced by mutant M188 (Glu^43^→Leu), which is consistent with the results from the mutants of PfMSP1-19 reported in other study [13]. There is a slight difference in the binding of mAb1E1 to the mutants by Western blot, which showed that the bindings to M176 and M188 were completely abolished. These results suggested that the residues of Leu^31 ^and Glu^43 ^were involved in the epitope of mAb1E1 on PfCP-2.9. In addition, the bindings of both mAb6G and mAbP5-W12 were abolished by M159 (Gln^14^→Gly) or M188 (Glu^43^→Leu), and reduced by M185 (Lys^40^→Ile). However, these antibodies showed no biological activity in GIA.

(3) A vital amino acid of Asn^15^, found in the present study, may be of critical importance for the correct folding of PfCP-2.9, since the substitution of Asn^15^→Arg on PfCP-2.9 reduced the binding of most mAbs and abolished the binding of both mAb6G and mAbP5-W12.

## Conclusions

This study focused on epitope mapping of PfCP-2.9 through generation of a panel of mAbs against PfCP-2.9 and a series of mutated proteins of PfCP-2.9 with single amino acid substitution. An inhibitory epitope on the AMA-1 (III) component, recognized by mAb7G, was associated with three amino acids, Phe^491^, Glu^511 ^and Arg^513^. The amino acid residues of Gln^14 ^and Glu^43 ^were involved in the formation of a non-inhibitory epitope recognized by two mAbs, mAb6G and mAbP5-W12. The blocking epitope of PfCP-2.9 recognized by mAb1E1 was the same as that described previously on PfMSP1-19. These findings provide precise functional epitopes on PfCP-2.9 and may prove useful in directing the design of more effective malaria vaccines.

## Competing interests

The authors declare that they have no competing interests.

## Authors' contributions

CL participated in the study design, experiments and data analysis, and wrote the manuscript; RW established the hybridoma cell lines and participated in the production and identification of mAbs; YW participated in the production and characterization of the mAbs; DZ participated in the study design and data analysis, ZH participated in the construction and expression of the mutated proteins; WP conceived of the study, participated in its design and data analysis, and contributed to manuscript writing and editing. All authors read and approved the final manuscript.
